# Involvement of bcl-2 and p21^waf1^ proteins in response of human breast cancer cell clones to Tomudex

**DOI:** 10.1038/sj.bjc.6690685

**Published:** 1999-09

**Authors:** L Orlandi, A Bearzatto, G Abolafio, C De Marco, M G Daidone, N Zaffaroni

**Affiliations:** Oncologia Sperimentale C, Department of Experimental Oncology, Istituto Nazionale per lo Studio e la Cura dei Tumori, via Venezian 1, Milan, 20133, Italy

**Keywords:** Tomudex, resistance, *bcl-2*, p21^wafl^, breast cancer cells

## Abstract

Mechanisms of resistance to Tomudex include increased thymidylate synthase activity, as well as reduced intracellular drug uptake and polyglutamation. However, little is known about other mechanisms of resistance, such as a possible protection against Tomudex-induced apoptosis mediated by *bcl-2*. We transfected the MDA-MB-435 human breast cancer cell line, which is characterized by a mutated *p53* gene, with cDNA of the *bcl-2* gene and generated two clones (MDA-bcl4 and MDA-bcl7) characterized by *bcl-2* expression twofold and fourfold that observed in the control cell clone (MDA^neo^). A concomitant overexpression of p21^wafl^ was also detected in the MDA-bcl7 clone. The MDA-bcl4 clone was three times more resistant to a 24-h Tomudex exposure than the MDA^neo^ clone, whereas the MDA-bcl7 clone was as sensitive to Tomudex as the control cell clone. A lower sensitivity of the MDA-bcl4 clone than MDA^neo^ and MDA-bcl7 clones to 5-fluorouracil and gemcitabine was also observed. No significant difference was noted in the susceptibility of clones to fludarabine and methothrexate. Basal levels of thymidylate synthase activity were superimposable in the three clones. Tomudex induced a marked accumulation of cells in the S phase in all the clones. However, an apoptotic hypodiploid DNA peak and the characteristic nuclear morphology of apoptosis were observed only in the MDA-bcl7 clone after exposure to Tomudex. No difference in the treatment-induced modulation of proteins involved in cell cycle progression (cyclin A, cdk2, pRB, E2F-1) and apoptosis (*bcl-2*, *bax*) was observed in the three clones. The only exception was that the expression of p21^wafl^ in the MDA-bcl4 clone was inducible at a Tomudex concentration much higher than that required to induce the protein in the other clones. Overall, the results indicate that bcl-2 and p21^wafl^ proteins concur in determining the cellular profile of sensitivity/resistance to Tomudex. © 1999 Cancer Research Campaign


*British Journal of Cancer* (1999) **81**(2), 252–260
© 1999 Cancer Research Campaign
Article no. bjoc.1999.0685

### Involvement of bcl-2 and p21$^{waf1}$ proteins in response of human breast cancer cell clones to Tomudex

**L Orlandi, A Bearzatto, G Abolafio, C De Marco, MG Daidone and N Zaffaroni**

Oncologia Sperimentale C, Department of Experimental Oncology, Istituto Nazionale per lo Studio e la Cura dei Tumori, via Venezian 1, 20133 Milan, Italy

**Summary** Mechanisms of resistance to Tomudex include increased thymidylate synthase activity, as well as reduced intracellular drug uptake and polyglutamation. However, little is known about other mechanisms of resistance, such as a possible protection against Tomudex-induced apoptosis mediated by *bcl-2*. We transfected the MDA-MB-435 human breast cancer cell line, which is characterized by a mutated *p53* gene, with cDNA of the *bcl-2* gene and generated two clones (MDA-bcl4 and MDA-bcl7) characterized by *bcl-2* expression twofold and fourfold that observed in the control cell clone (MDA$^{neo}$). A concomitant overexpression of p21$^{wafl}$ was also detected in the MDA-bcl7 clone. The MDA-bcl4 clone was three times more resistant to a 24-h Tomudex exposure than the MDA$^{neo}$ clone, whereas the MDA-bcl7 clone was as sensitive to Tomudex as the control cell clone. A lower sensitivity of the MDA-bcl4 clone than MDA$^{neo}$ and MDA-bcl7 clones to 5-fluorouracil and gemcitabine was also observed. No significant difference was noted in the susceptibility of clones to fludarabine and methothrexate. Basal levels of thymidylate synthase activity were superimposable in the three clones. Tomudex induced a marked accumulation of cells in the S phase in all the clones. However, an apoptotic hypodiploid DNA peak and the characteristic nuclear morphology of apoptosis were observed only in the MDA-bcl7 clone after exposure to Tomudex. No difference in the treatment-induced modulation of proteins involved in cell cycle progression (cyclin A, cdk2, pRB, E2F-1) and apoptosis (*bcl-2*, *bax*) was observed in the three clones. The only exception was that the expression of p21$^{wafl}$ in the MDA-bcl4 clone was inducible at a Tomudex concentration much higher than that required to induce the protein in the other clones. Overall, the results indicate that bcl-2 and p21$^{wafl}$ proteins concur in determining the cellular profile of sensitivity/resistance to Tomudex.

© 1999 Cancer Research Campaign

**Keywords:** Tomudex; resistance; *bcl-2*; p21$^{wafl}$; breast cancer cells

Tomudex (TDX), a quinazoline folate analogue, is a pure inhibitor of thymidylate synthase (TS), the enzyme which catalyses the methylation of deoxyuridine monophosphate, using 5,10-methylene-tetrahydrofolate as a methyl donor, to deoxythymidine monophosphate, an essential precursor of DNA synthesis (Jackman et al, 1991*a*; Jackman and Calvert, 1995; Rustum et al, 1997). TDX structurally resembles the physiological folates and requires an active transmembrane transport system. Specifically, the drug can be internalized via both the reduced folate carrier (which also transports methotrexate) and the membrane-associated folate binding protein (Westerhof et al, 1995; Pinard et al, 1996). The drug is metabolized to polyglutamate forms, which are more potent as inhibitors of TS and are more slowly effluxed from cells than the unmetabolized monoglutamate compound (Jackman et al, 1993). Panadero et al (1995) showed that TDX induced extensive fragmentation of genomic DNA and newly synthesized DNA in HCT-8 ileocaecal adenocarcinoma cells and that the effect was correlated with an increase in the deoxyuridine triphosphate pool and a concomitant decrease in the deoxythymidine triphosphate pool.

TDX has shown activity in a variety of cell lines of different human tumour histotypes (Jackman et al, 1995*a*; Kelland et al, 1995) and in animal tumour models (Jackman et al, 1991*b*). The drug has also demonstrated clinical benefits in terms of higher response rates and lower toxicity than obtained with 5-fluorouracil (5-FU) plus leucovorin in patients with advanced colorectal cancer (Cunningham et al, 1995). It is now currently used in the clinical treatment of such tumour type, alone or in combination with other agents including 5-FU (infusion and bolus), platinum compounds and topoisomerase inhibitors (Van Cutsem, 1997; Blackledge, 1998). Moreover, TDX has demonstrated encouraging activity against ovarian, non-small-cell lung, pancreatic and breast cancer (Smith et al, 1996; Judson, 1997).

Since the major limitation to the successful use of chemotherapeutic agents is the development of clinical resistance, it is important to identify the potential mechanisms of resistance to TDX in tumour cells. Several biochemical determinants of resistance to TDX have been investigated, including decreased drug uptake by the reduced folate/methotrexate carrier, impaired intracellular polyglutamation and increased TS levels (Freemantle et al, 1995; Jackman et al, 1995*b*; Lu et al, 1995; Drake et al, 1996). Conversely, little is known about other potential mechanisms of resistance to TDX, such as a possible protection against TDX-induced apoptosis mediated by *bcl-2*. To address this issue, we transfected the *p53* mutant MDA-MB-435 breast cancer cell line with cDNA of the *bcl-2* gene and assessed the sensitivity profile to TDX in two selected clones with different expression of bcl-2 and cyclin-dependent kinase (cdk) inhibitor p21$^{wafl}$ proteins. A different sensitivity to TDX, in terms of cell survival and susceptibility to undergo apoptosis, was observed in the different clones.

*Received 12 October 1998*
*Revised 23 February 1999*
*Accepted 25 February 1999*

*Correspondence to:* N Zaffaroni

In order to elucidate such differences, we analysed the effects of TDX on proteins involved in the control of cell cycle progression (mainly G1 to S phase) and apoptosis. In parallel, sensitivity of cell clones to other antimetabolites or drugs with different action mechanisms was also assessed.

### MATERIALS AND METHODS

### Cell lines and transfection procedures

The MDA-MB-435 human breast cancer cell line used in the study is characterized by a mutated *p53* gene (266GGA→GAA) (O'Connor et al, 1997). The *bcl-2* gene under the control of the SSFV promoter inserted into the native LTR-SV neo vector (kindly provided by Dr SJ Korsmeyer, Washington University, St Louis, MO, USA) was introduced into MDA-MB-435 cells using Lipofectamine (Gibco-BRL, Gaithersburg, MD, USA) according to the manufacturer's protocol. MDA-MB-435 cells ($4 \times 10^5$) were plated in 100-mm dishes; the next day they were washed with phosphate-buffered saline (PBS) and overlaid with serum-free Dulbecco's modified Eagle's medium (DMEM) Ham's F-12 (Gibco) containing 10 µg linearized bcl-2 expression or control vector DNA and 20 µl Lipofectamine. After 16 h, medium was replaced with DMEM/Ham's F-12 containing 10% fetal bovine serum (FBS; Gibco) for 5 h, followed by incubation in DMEM/Ham's F-12 containing 5% FBS for 24 h. Stable transfectants were selected in the presence of 900 µg $ml^{-1}$ G418 (Geneticine, Gibco). After 15–18 days, 40 resistant colonies containing bcl-2-expressing vector or the vector alone were harvested and tested for bcl-2 expression by Western blot analysis. Overall, three clones were selected: MDA$^{neo}$ (control vector), MDA-bcl4 and MDA-bcl7 (bcl-2 transfectants).

### Chemicals

Doxorubicin (Pharmacia-Upjohn, Uppsala, Sweden) was dissolved in normal saline solution. Taxol (Sigma Chemical Co., St Louis, MO, USA) was stored as a 200 µmol $µl^{-1}$ stock solution in dimethyl sulphoxide and then reconstituted and diluted in sterile water to obtain a solvent concentration of less than 0.25%. Fludarabine (9-β-arabinofuranosyl-2-fluoroadenine-5′ monophosphate, Fludara, kindly supplied by Shering, Berlin, Germany) was reconstituted in PBS. Gemcitabine (2′,2′-difluorodeoxycytidine, kindly supplied by Eli Lilly, Indianapolis, IN, USA) was reconstituted in sterile water. 5-FU (Fluoro-uracile, Roche, Basel, Switzerland) and methotrexate (Lederle, Cyanamid, Aprilia (LT), Italy) were diluted in sterile water. TDX (ZD1694, [*N*-(5-[*N*-(3,4-dihydro-2-methyl-4-oxoquinazolin-6-ylmethyl)-*N*-methylamino]-2-thenoyl)-L-glutamic acid], kindly supplied by Zeneca, Pharmaceuticals, Macclesfield, UK), was dissolved in 0.1 M sodium bicarbonate, pH 8.3, at a concentration of 200 µg $ml^{-1}$ and diluted in DMEM/Ham'S F-12 medium.

### Cell proliferation studies

The MDA-MB-435 parental cell line, the MDA$^{neo}$ clone (transfected with LTR-SV neo vector alone) and bcl-2 transfectants (MDA-bcl4 and MDA-bcl7) were cultured in DMEM/Ham's F-12 medium lacking G418, supplemented with 5% FBS. After harvesting in the logarithmic growth phase, cells were seeded in 6-well plates and treated with varying doses of taxol, fludarabine, gemcitabine, 5-FU, methotrexate and TDX for 24 h, or doxorubicin for 1 h. Exposure times to different drugs were chosen according to the treatment times used in previous studies (Silvestrini et al, 1993). At the end of treatment, adherent cells were washed with PBS and incubated at 37°C in a 5% carbon dioxide humidified atmosphere for 3 days. Cells were then trypsinized and counted in a particle counter (Coulter Counter, Coulter Electronics, Luton, UK). The percentages of adherent viable cells were determined by the Trypan blue dye exclusion test. Viability always exceeded 95%. Each experimental sample was run in triplicate. The results were expressed as the total number of adherent cells in treated samples compared with control samples. In vitro activities of different drugs were expressed in terms of concentrations able to inhibit cell proliferation by 50% ($IC_{50}$).

### TS catalytic activity assay

The assay proposed by Van der Wilt et al (1992) was used with some modifications. Briefly, $10 \times 10^6$ cells were suspended in 0.8 ml of ice-cold assay buffer containing 200 mM Tris–HCl, 20 mM β-mercaptoethanol, 100 mM NaF and 15 mM cytidine-5′-monophosphate (pH 7.4). Cells were then sonicated and centrifuged at 12 000 rpm. The supernatants containing the enzyme (enzyme suspensions) were split into two parts for protein content determination and TS catalytic activity assay. The TS catalytic activity assay was carried out with 10 µl of enzyme suspensions (in different dilutions), 5 µl $N^5$,$N^{10}$-methylene-tetrahydrofolate and 10 µl of [5-$^3$H]-2′-deoxyuridine-5′-monophosphate (10-µM final concentration). Reaction mixtures were incubated for 30 min at 37°C and stopped by addition of 50 µl ice-cold 35% trichloroacetic acid and 250 µl 10% neutral activated charcoal. After centrifugation, radioactivity present in 150 µl of supernatant was determined by liquid scintillation counting. TS activity was expressed as $^3$H release (pmol $h^{-1}$ $10^{-6}$ cells) produced during the conversion of [$^3$H]deoxyuridine monophosphate to $^3H_2O$.

### Flow cytometric analysis

Immediately after a 24-h TDX exposure, or after an additional 8 and 24 h in drug-free medium, samples of $1 \times 10^6$ cells were fixed in 70% ethanol. Before analysis, cells were washed in PBS and stained with a solution containing 50 µg $ml^{-1}$ propidium iodide, 50 mg $ml^{-1}$ RNAase, and 0.05% NP40 for 30 min at 4°C and then analysed with a FACScan flow cytometer (Becton Dickinson, Sunnyvale, CA, USA). An aliquot of each sample was also analysed under fluorescence microscopy for evaluation of nuclear morphology. The cell cycle distribution was evaluated on DNA plots by CellFit software according to the SOBR model (Becton Dickinson).

### Immunohistochemistry

After trypsinization of exponentially growing clones, samples of cell suspensions were seeded onto glass slides, air-dried and immediately fixed in formalin for 3 min. Fixed cells were rehydrated with PBS and labelled with the monoclonal antibody anti-p21$^{waf1}$ (clone DCS 60.2; Neomarkers, Freemont, CA, USA). Antibody binding to cells was evidenced by the biotin–streptavidin alkaline phosphatase technique.

© 1999 Cancer Research Campaign 

### Cell lysis and immunoblotting

Cells were lysed in 1% Nonidet P-40, prepared in PBS containing 10 μg $ml^{-1}$ leupeptin, 10 μg $ml^{-1}$ aprotinin, 1 mM AEBSF, 1 mM $Na_3VO_4$, 1mM $NaPPO_4$ and 10 mM NaF. Cell lysates were clarified (15 min, 15 000 rpm), and the resultant supernatants were used for protein analysis. Total cellular lysate (100 μg) was dissolved in 2 × sample loading buffer (2% sodium dodecyl sulphate (SDS), 5% 2-mercaptoethanol, 20% glycerol, 60 mM m Tris, pH 6.8, and 0.0025% bromophenol), separated on 12% SDS-polyacrylamide gel and transferred to nitrocellulose. Filters were blocked in PBS with 5% skim milk and then incubated overnight with the primary monoclonal antibody anti-pRb (retinoblastoma protein), anti-Bax, anti-Bcl-2, anti E2F-1, anti-cyclin A and anti-cdk2 (Santa Cruz Biotechnology, Santa Cruz, CA, USA), anti-$p21^{wafl}$ (Oncogene Science, Cambridge, MA, USA) or anti-TS (kindly supplied by Dr PG Johnston, Queen's University, Belfast, Ireland). Filters were then incubated with the secondary antibody antimouse or antirabbit Ig horseradish peroxidase-linked whole antibody (Amersham, Buckinghamshire, UK). Bound antibody was detected using the enhanced chemiluminescence Western blotting detection system (Amersham). An anti-PCNA (proliferating cell nuclear antigen) monoclonal antibody (Santa Cruz Biotechnology) was used on each blot to ensure equal loading of protein on the gel.

### RESULTS

### Cell clone characteristics and sensitivity to drugs

The parental MDA-MB-435 cell line and the control transfectant $MDA^{neo}$ clone showed very low and similar levels of bcl-2 expression, whereas MDA-bcl4 and MDA-bcl7 clones were characterized by bcl-2 expression twofold and fourfold (as evaluated by densitometric analysis of immunoblotting) that observed in $MDA^{neo}$ and MDA-MB-435 cells (Figure 1). No difference was observed in any of the three clones and the parental cell line as regards the expression of p53 and bax proteins. Conversely, an overexpression of $p21^{wafl}$ protein was observed in MDA-bcl7 (Figure 1). Specially, the level of $p21^{wafl}$ in the MDA-bcl7 clone was twofold that observed in $MDA^{neo}$ cells. $p21^{wafl}$ results obtained by Western blotting were also confirmed by immunohistochemistry (Figure 2). Analysis of the logarithmic growth rate of bcl-2-transfected clones and of the clone transfected with the control vector indicated a doubling time of approximately 20 h for all the cell lines (Table 1). Moreover, similar basal catalytic activities of TS were observed in $MDA^{neo}$, MDA-bcl4 and MDA-bcl7.

As regards sensitivity to different anticancer agents (Table 2), the three clones showed similar susceptibility to the antimetabolite fludarabine and drugs with other mechanisms of action such as doxorubicin and taxol, as indicated by the superimposable $IC_{50}$ values. The MDA-bcl4 clone was slightly, although not significantly, less sensitive than $MDA^{neo}$ and MDA-bcl7 clones to methotrexate. Moreover, the MDA-bcl4 clone was significantly more resistant to 5-FU, gemcitabine ($P$ < 0.025) and TDX ($P$ < 0.01) than the other two clones.

### Effects of TDX on the cell cycle

DNA flow cytometric analysis was performed to determine whether cell cycle perturbations could explain the different cytotoxic activity of TDX in the three clones (Table 3). For $MDA^{neo}$ and MDA-bcl7, TDX was used at a concentration of 8 μM, which is the $IC_{50}$ of the two clones. For MDA-bcl4, besides the 8 μM concentration, the specific $IC_{50}$ of the clone (25 μM) was also used. Immediately after a 24-h treatment with 8 μM of TDX, a very slight accumulation of cells in the S phase was observed in all three clones. Such accumulations became more evident 48 h after beginning the TDX treatment, when most of the cells were arrested in the S phase. The extent of the accumulation was superimposable in the clones independently of the different cytotoxic effects induced by the drug. Moreover, a marked decrease in the G2/M cell fraction, consistent with an inhibition of dTTP synthesis induced by TDX, was observed in $MDA^{neo}$ and MDA-bcl4 clones. In the MDA-bcl4 clone, exposure to 25 μM of TDX induced a slight accumulation of cells in the G0/1 phase 24 h and 32 h after beginning the TDX treatment, followed by a marked accumulation of cells in the S phase, to an extent similar to that observed with the 8-μM TDX treatment.

To investigate at a molecular level possible differences in the events that regulate progression through the S phase, we analysed the effects of 8 μM TDX treatment on pRb, E2F-1, cyclin A and cdk2 protein expression. An increase in E2F-1, cyclin A and cdk2 levels was observed in all cell clones, whereas no treatment-induced modulation of pRb expression and phosphorylation was observed (Figure 3).

### Induction of apoptosis by TDX

Flow cytometric analysis of the three clones exposed to 8 μM TDX showed that 48 h after beginning the treatment, approximately 14% of MDA-bcl7 cells exhibited a hypodiploid DNA content. Such a pre-G1 apoptotic cell peak was not observed in MDA-bcl4 or in cells transfected with the control vector (Figure 4). In addition, evaluation by fluorescence microscopy of cell clones stained with propidium iodide confirmed the presence of cells with the characteristic nuclear morphology of apoptosis only in the

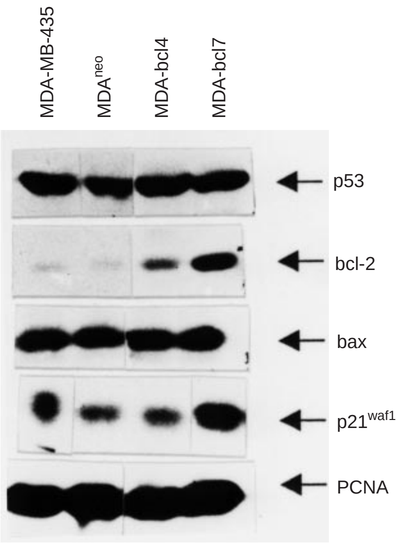

**Figure 1** A representative experiment illustrating the basal expression of p53, bcl-2, bax and $p21^{wafl}$ proteins in MDA-MB-435 (parental cell line), $MDA^{neo}$ (control vector) and MDA-bcl4 and MDA-bcl7 (the two selected bcl-2 transfectant clones). PCNA was used as a control for correct loading

© 1999 Cancer Research Campaign

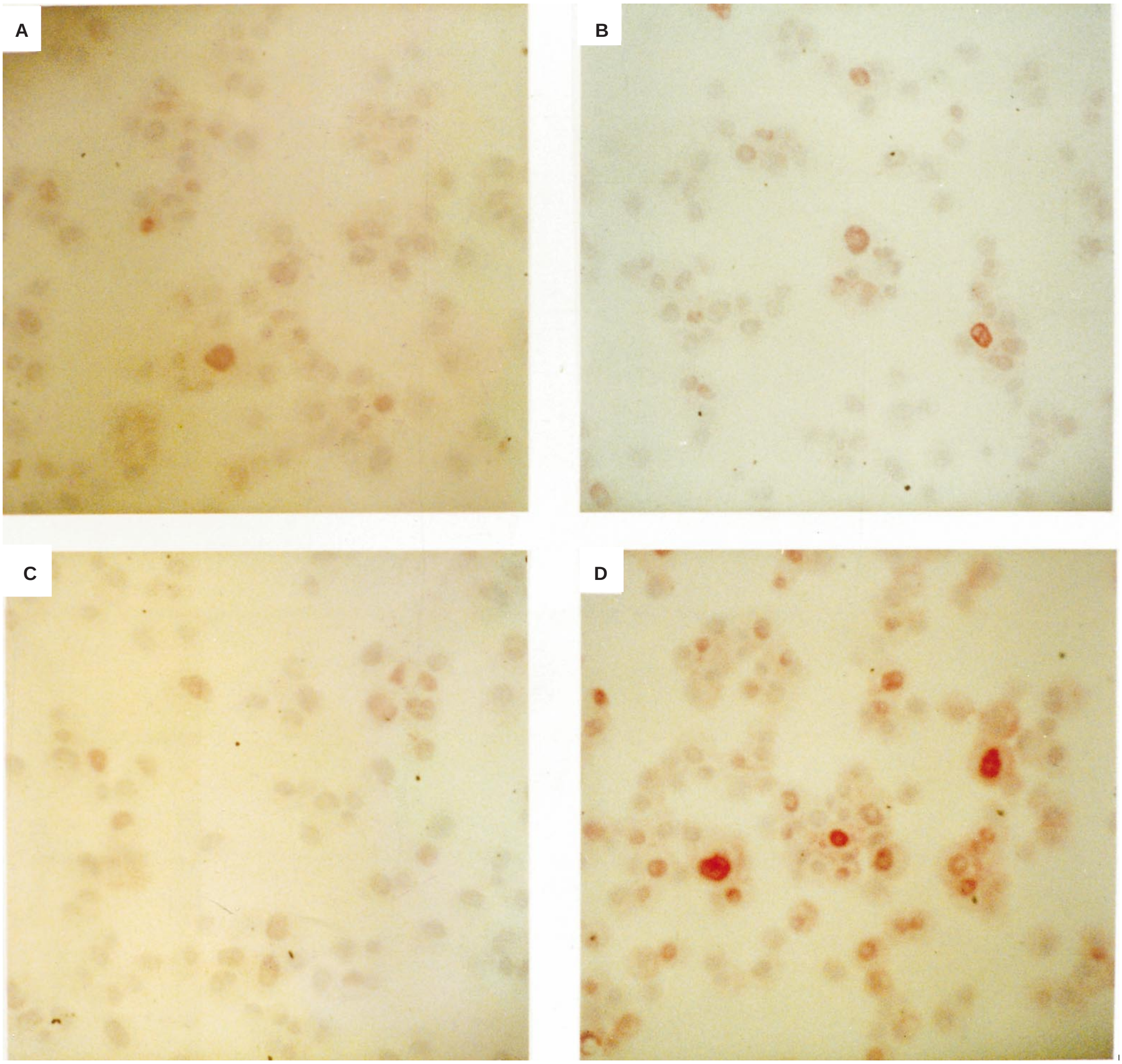

**Figure 2** Immunohistochemical detection of p21$^{waf1}$ expression in MDA-MB-435 (**A**), MDA$^{neo}$ (**B**), MDA-bcl4 (**C**) and MDA-bcl7 (**D**)

MDA-bcl7 clone (Figure 5). In order to elucidate the pathway of TDX-induced programmed cell death, the effect of treatment on proteins involved in the onset of apoptosis was assessed. As shown in Figure 6, no appreciable modulation of bax or bcl-2 protein expression, or in the bcl-2 phosphorylation status, was observed in any of the three clones. Conversely, an induction of p21$^{waf1}$ expression was found after exposure to 8 μM TDX in MDA$^{neo}$ and MDA-bcl7 but not in the MDA-bcl4 clone. Such an increase was maximal at 48 h after beginning the TDX treatment. In the MDA-bcl4 clone, a marked induction of the p21$^{waf1}$ protein became apparent when cells were treated with the highest (25 μM) TDX concentration (Figure 7).

### Effect of TDX on TS expression

Finally, to verify whether or not TDX differentially affected the expression of its target enzyme, thus influencing cellular sensitivity to the drug, we analysed the expression in TS protein after treatment with 8 μM TDX. As shown in Figure 8, an increase of TS expression was appreciable in all cell clones to a similar extent.

### DISCUSSION

TDX is a specific inhibitor of TS and has demonstrated its activity in many solid tumours in experimental systems. Moreover, among novel TS inhibitors, TDX is furthest along in its clinical development (Van Cutsem, 1997). Due to increasing clinical interest in the drug, it is important to identify possible mechanisms of inherent or acquired resistance, other than the well-known classical biochemical mechanisms of resistance to antimetabolites (Kinsella et al, 1997).

The present study investigated the role of bcl-2 and p21$^{waf1}$ proteins in the response of human breast cancer cell clones to TDX. The MDA-MB-435 human breast cancer cell line was transfected

© 1999 Cancer Research Campaign

**Table 1** Characteristics of cell clones

| Clone | Doubling time (h) | Thymidylate synthase activity[a] (pmol h$^{-1}$ 10$^{-6}$ cells) |
|---|---|---|
| | | 10 μM dUMP |
| MDA$^{neo}$ | 22 | 1994 ± 231 |
| MDA-bcl4 | 20 | 2146 ± 212 |
| MDA-bcl7 | 22 | 2647 ± 271 |

[a] Data represent mean values ± s.d. of three independent experiments.

**Table 2** Sensitivity of breast cancer cell clones to different drugs

| | IC$_{50}$ (μM) | | |
|---|---|---|---|
| | MDA$^{neo}$ | MDA-bcl4 | MDA-bcl7 |
| Doxorubicin[a] | 1.20 ± 0.22 | 1.00 ± 0.12 | 1.00 ± 0.18 |
| Taxol[b] | 0.046 ± 0.0028 | 0.043 ± 0.0037 | 0.036 ± 0.0041 |
| Fludarabine[b] | 0.62 ± 0.12 | 0.67 ± 0.13 | 0.66 ± 0.15 |
| Gemcitabine[b] | 0.30 ± 0.80 | 0.57 ± 0.10[c] | 0.47 ± 0.16 |
| 5-Fluorouracil[b] | 36.1 ± 7.00 | 60.9 ± 3.00[d] | 35.7 ± 1.00 |
| Methotrexate[b] | 6.30 ± 0.50 | 10.2 ± 1.10 | 4.84 ± 0.60 |
| Tomudex[b] | 7.60 ± 2.00 | 25.4 ± 4.00[e] | 7.7 ± 2.60 |

[a]1-h exposure. [b]24-h exposure. [c]$P < 0.025$, Student's *t*-test with respect to MDA$^{neo}$. [d]$P < 0.025$, Student's *t*-test with respect to MDA$^{neo}$ and MDA-bcl7. [e]$P < 0.01$, Student's *t*-test with respect to MDA$^{neo}$ and MDA-bcl7. Data represent mean values ± s.d. of at least three independent experiments.

with cDNA of the *bcl*-2 gene. Two clones (MDA-bcl4 and MDA-bcl7), both characterized by bcl-2 expression respectively twofold and fourfold that observed in the control cell clone tranfected with c-DNA of the neomycin-resistance gene alone (MDA$^{neo}$), were generated. The three clones were similar in growth profile, basal TS activity and expression, and expression of p53 and bax, whereas in the MDA-bcl7 clone an induction of p21$^{wafl}$ concomitant to bcl-2 overexpression was observed.

Analysis of the chemosensitivity profiles of the three cell clones showed a similar sensitivity towards the antimetabolite fludarabine. However, the MDA-bcl4 clone was more resistant than MDA$^{neo}$ and MDA-bcl7 clones to other antimetabolites such as gemcitabine, 5-FU and methotrexate, and particularly resistant (threefold) to TDX. Overall, it emerged that transfection of cells with bcl-2 reduced sensitivity to TDX and that such resistance was not evident in cells that concomitantly overexpressed p21$^{wafl}$. Such results are in agreement with the work of Fisher et al (1993), who reported that Burkitt's lymphoma cells transfected with bcl-2 showed an increased cell viability and a concomitant reduction of apoptosis, compared to control cells, after treatment with the antimetabolite fluorodeoxyuridine and quinazoline-based TS inhibitors such as CB3717 and ICI M247496. Similarly to our results, the authors also showed that cell cycle and TS level and activity were not altered by transfection of lymphoma cells with bcl-2.

The results of cross-resistance studies support the possibility that in the MDA-bcl4 clone there is a polyglutamation defect or a reduced uptake of the drug, since methotrexate, which shares the same carrier with TDX and also undergoes polyglutamation, showed a reduced activity in the clone. However, such a hypothesis does not completely explain the higher resistance to TDX than to methotrexate observed in the clone. Moreover, we can exclude that the partial cross-resistance of TDX and 5-FU in the MDA-bcl4 clone is due to TS alterations, since in the clone the basal activity of TS was similar to that of MDA$^{neo}$ and MDA-bcl7.

A finding that deserves further investigation is that TDX resistance was completely overcome in the MDA-bcl7 clone overexpressing concomitantly bcl-2 and p21$^{wafl}$. p21$^{wafl}$, which was originally identified as an inhibitor of cdk2, cdk4 and cdc2 kinase complexes (Xiong et al, 1993), is a transcriptional target of p53 and is strongly induced by DNA damage in cells expressing functional p53 (El-Deiry et al, 1994). However, its activation can also occur independently of p53 (Russo et al, 1993; Sheikh, 1994). In the presence of functional pRb, p21$^{wafl}$ inhibits the activity of cyclin D–cdk4, cyclin A–cdk2 and cyclin E–cdk2 complexes, thereby increasing the level of hypophosphorylated pRb, which in turn associates with E2F-1 and arrests the cells in G1 phase (Chellappan et al, 1991; Ikeda et al, 1996). Moreover, p21$^{wafl}$ is involved in tumour growth suppression and cellular response to DNA damage (Chen et al, 1995; Garthenhaus et al, 1995). We therefore investigated whether differences in TDX sensitivity could be supported by differences in cell cycle perturbations

**Table 3** Cell cycle perturbations induced by Tomudex

| | Time[a] | | | | | | | | |
|---|---|---|---|---|---|---|---|---|---|
| | 24 h | | | 32 h | | | 48 h | | |
| | G0/1 | S | G2/M | G0/1 | S | G2/M | G0/1 | S | G2/M |
| MDA$^{neo}$ | | | | | | | | | |
| Control | 55 ± 3 | 30 ± 1 | 15 ± 1 | 53 ± 1 | 32 ± 1 | 15 ± 2 | 50 ± 1 | 33 ± 2 | 17 ± 1 |
| Tomudex 8 μM | 52 ± 7 | 42 ± 9 | 6 ± 1 | 50 ± 7 | 39 ± 5 | 11 ± 3 | 16 ± 1 | 71 ± 5 | 13 ± 5 |
| MDA-bcl4 | | | | | | | | | |
| Control | 55 ± 7 | 28 ± 3 | 17 ± 5 | 53 ± 1 | 30 ± 3 | 17 ± 3 | 47 ± 5 | 32 ± 2 | 21 ± 7 |
| Tomudex 8 μM | 53 ± 8 | 38 ± 11 | 9 ± 2 | 51 ± 13 | 38 ± 10 | 11 ± 3 | 24 ± 6 | 67 ± 11 | 9 ± 11 |
| Tomudex 25 μM | 67 ± 7 | 25 ± 9 | 8 ± 1 | 67 ± 7 | 25 ± 6 | 8 ± 1 | 24 ± 9 | 65 ± 14 | 11 ± 5 |
| MDA-bcl7 | | | | | | | | | |
| Control | 56 ± 1 | 27 ± 4 | 17 ± 2 | 46 ± 4 | 38 ± 7 | 16 ± 3 | 50 ± 4 | 31 ± 4 | 18 ± 2 |
| Tomudex 8 μM | 50 ± 10 | 39 ± 8 | 11 ± 3 | 43 ± 13 | 38 ± 6 | 19 ± 2 | 19 ± 7 | 69 ± 4 | 12 ± 2 |

[a]Calculated from the beginning of Tomudex treatment. Data represent mean values ± s.d. of three independent experiments.

© 1999 Cancer Research Campaign

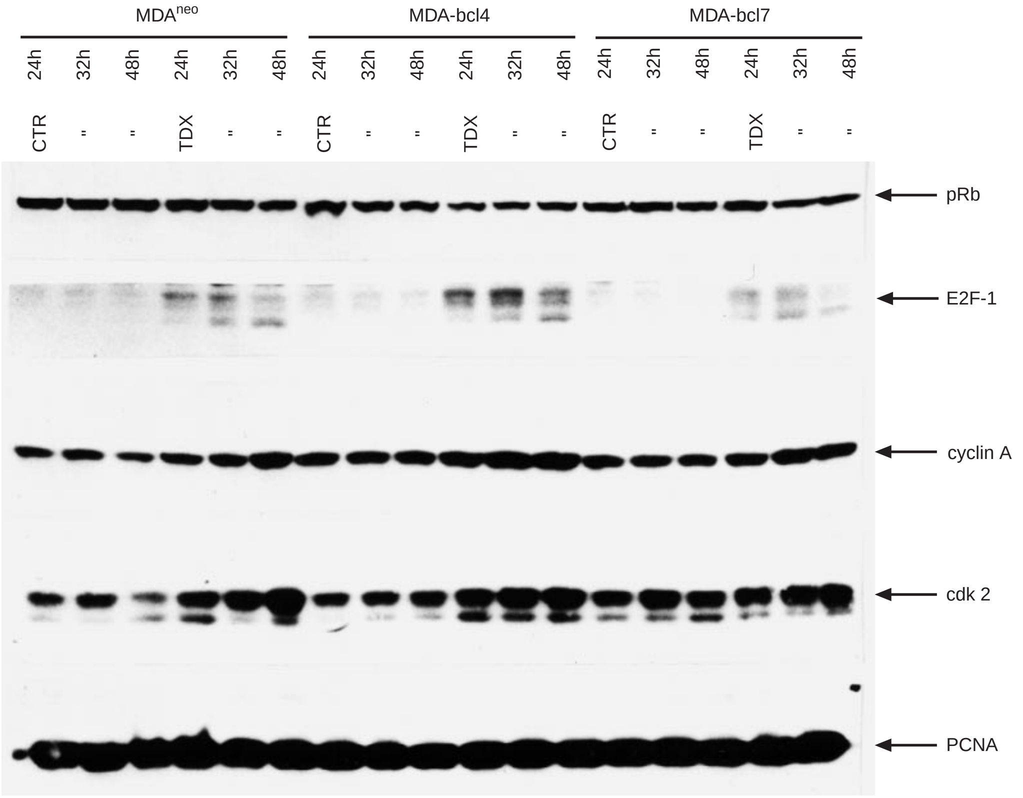

**Figure 3** A representative experiment illustrating the temporal effects of TDX treatment on the expression of proteins involved in control of the S phase in MDA$^{neo}$, MDA-bcl4 and MDA-bcl7 cells. The three clones were incubated with solvent (control, CTR) or 8 $\mu$M TDX for 24 h. At the end of the treatment, cells were washed with PBS, immediately collected (in Figure indicated as 24 h) or incubated for an additional 8 h and 24 h in drug-free medium (in Figure indicated as 32 h and 48 h from the beginning of TDX treatment). Western blots were probed with monoclonal antibodies for pRb, E2F-1, cyclin A and cdk2. PCNA was used as control for correct loading

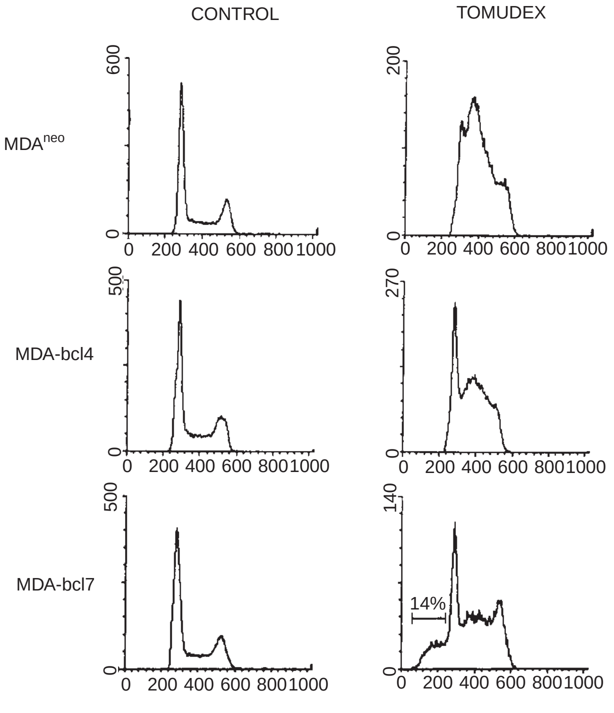

**Figure 4** Cell cycle analyses on MDA$^{neo}$, MDA-bcl4 and MDA-bcl7 cells 48 h after beginning a 24-h TDX treatment (8 $\mu$M concentration) and in solvent-treated control cells. The percentage of the pre-G1 population is reported in the bottom right-hand corner for MDA-bcl7

induced by TDX treatment. The TS inhibitor caused a marked accumulation of cells in the S phase, which was superimposible in all three clones. Moreover, in the MDA-bcl4 clone, TDX resistance could not be ascribed to a different interference of the drug on the cell cycle, since comparable S phase cell accumulations were observed after treatment with two drug concentrations (8 and 25 $\mu$M), which induced different cytotoxic effects.

At a molecular level, TDX induced increases in E2F-1, cyclin A and cdk2 levels as cells progressed and accumulated in the S phase, whereas pRb expression and phosphorylation did not show any change as a function of TDX treatment. Although a direct comparison between the data obtained in the different clones by using a non-equitoxic TDX concentration cannot be made, the superimposable patterns of protein modulation observed suggest that the levels of such proteins are not responsible for the different sensitivity to TDX but that they merely reflect a drug-induced cell cycle perturbation which is consistent in the three clones. A time-dependent increase in TS protein was similarly observed in all the three clones after TDX exposure. TS overexpression could be due to the transactivation by E2F-1 of the TS gene, which is required for entry of cells in the S phase (Sherr, 1996), or, alternatively, to a cellular effort to overcome the cytotoxic stress induced by TDX by increasing the production of its target enzyme.

Since bcl-2 (Kroemer, 1997) and p21$^{waf1}$ (El-Deiry, 1994) are involved in the apoptotic process, a possibility is that a different

*© 1999 Cancer Research Campaign*

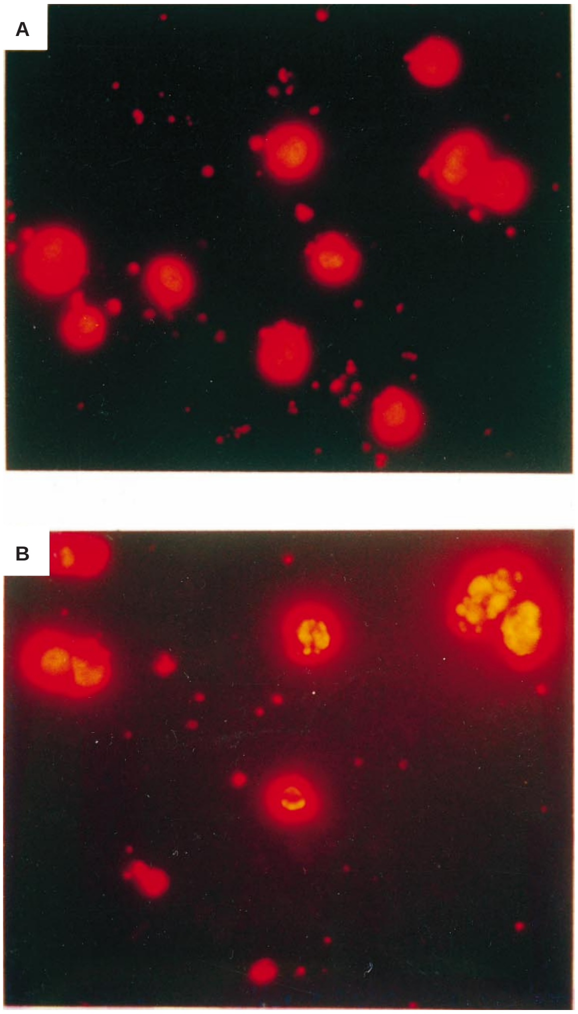

**Figure 5** Propidium iodide staining of MDA-bcl7 cells treated with solvent alone (**A**) or with 8 μM TDX (**B**) and collected 48 h after beginning a 24-h TDX treatment. Whole cells were collected, stained with propidium iodide and viewed under the fluorescence microscope induction and regulation of apoptosis could be responsible for TDX resistance in the MDA-bcl4 clone and for restoring TDX sensitivity in the MDA-bcl7 clone. Flow cytometric analysis evidenced a pre-G1 apoptotic cell peak only in the MDA-bcl7 clone 48 h after beginning TDX treatment, and morphologic analysis confirmed the presence of apoptotic cells only in this clone. However, since the analysis was performed only at a constant TDX concentration, which produced different cytotoxic effects in the two cell clones, on the basis of our data it is not possible to establish that a lower susceptibility to undergo apoptosis was responsible for resistance to TDX in the MDA-bcl4 clone.

As regards the effect of TDX on the expression of proteins involved in the onset of apoptosis, no change was observed in bcl-2 and bax compared to basal levels of control, whereas an induction of $p21^{wafl}$ was observed in the two clones with similar sensitivity to TDX, $MDA^{neo}$ and MDA-bcl7, but not in the MDA-bcl4-resistant clone. In fact, in the latter clone, a higher TDX concentration was necessary to produce an appreciable induction of $p21^{wafl}$. We could hypothesize that enhanced basal expression and inducibility of $p21^{wafl}$ was able to overcome the anti-apoptotic effect of bcl-2 and to restore the sensitivity to TDX in MDA-bcl7.

Our results are in agreement with the findings of Li et al (1997), who showed that in SaOs-2 osteosarcoma cells, lacking p53 and pRb, overexpression of $p21^{wafl}$ increased sensitivity to TDX, methotrexate and doxorubicin by increasing the extent of apoptosis. The overexpression of $p21^{wafl}$ reduced cyclin A-associated kinase activity and resulted in inhibition of phosphorylation of E2F-1 and increased E2F-1 binding activity. Such events lead to an enhanced S–G2 cell cycle delay and increased susceptibility to apoptosis. Since in our experimental models no effect of $p21^{wafl}$ overexpression on the duration of the S phase block was observed, we hypothesize that the pathway described by Li et al (1997) could be different from that followed by our clones.

Overall, our results suggest that bcl-2 and $p21^{wafl}$ proteins concur in determining the profile of sensitivity/resistance of MDA-MB-435 breast cancer cells to TDX. The reacquisition of TDX sensitivity in the MDA-bcl7 clone could be mediated, at least

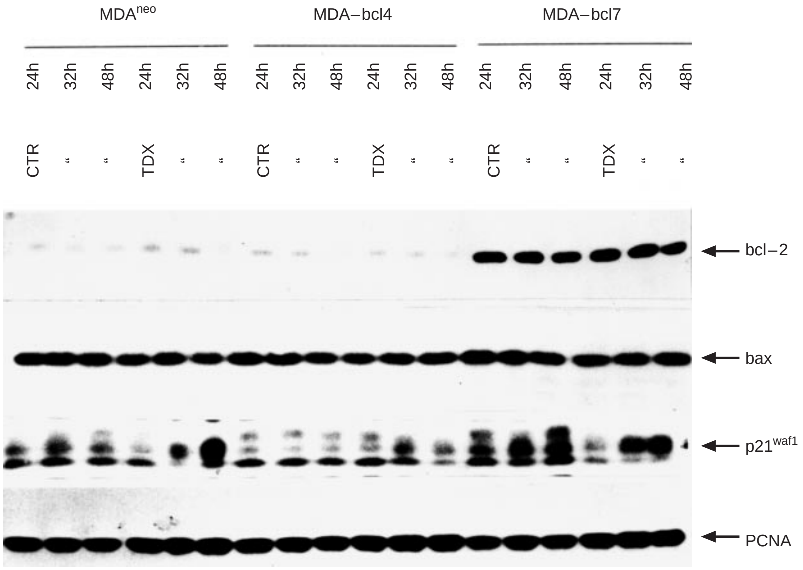

**Figure 6** A representative experiment illustrating the temporal effects of TDX treatment on the expression of proteins involved in the onset of apoptosis in $MDA^{neo}$, MDA-bcl4 and MDA-bcl7 cells. Treatments and samples as indicated in Figure 3. Western blots were probed with monoclonal antibodies for bcl-2, bax and $p21^{wafl}$. PCNA was used as a control for correct loading

 *© 1999 Cancer Research Campaign*

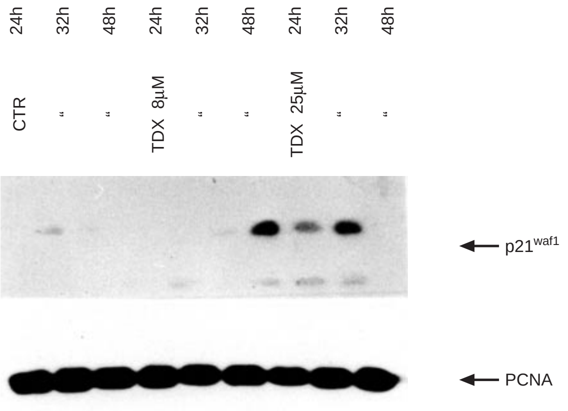

**Figure 7** A representative experiment illustrating the effect of TDX on p21$^{wafl}$ expression in MDA-bcl4 cells. Cells were incubated with solvent (control, CTR), 8 μM TDX or 25 μM TDX for 24 h. At the end of the treatment, cells were washed with PBS, immediately collected (in Figure indicated as 24 h) or incubated for an additional 8 h and 24 h in drug-free medium (in Figure indicated as 32 h and 48 h from the beginning of TDX treatment). PCNA was used as control for correct loading in part, by overexpression of p21$^{wafl}$ and susceptibility of cells to undergo apoptosis. Several studies have shown that overexpression of p21$^{wafl}$ alone can induce apoptosis following transfection (Sheikh et al, 1995; Prabhu et al, 1996). However, the role of p21$^{wafl}$ in the apoptosis process is still controversial. Studies are warranted to elucidate the molecular mechanisms by which bcl-2 and p21$^{wafl}$ are involved in TDX cytotoxic activity and apoptosis.

### ACKNOWLEDGEMENTS

The study was supported by grants from the Associazione Italiana per la Ricerca sul Cancro and the National Research Council. The authors thank Mrs. B. Canova and Miss B Johnston for editorial assistance. The authors also thank the EORTC Preclinical Tumor Models Group for encouraging the study.

### REFERENCES

Blackledge G (1998) New developments in cancer treatment with the novel thymidylate synthase inhibitor raltitrexed ('Tomudex'). *Br J Cancer* **77**: 29–37

Chellappan SP, Hiebert S, Mudryj M, Horowitz JM and Nevins JR (1991) The E2F transcription factor is a cellular target for Rb protein. *Cell* **65**: 1053–1061

Chen YQ, Cipriano SC, Arenkiel JM and Miller FR (1995) Tumor suppression by p21$^{wafl}$. *Cancer Res* **55**: 4536–4539

Cunningham D, Zalcberg JR, Rath U, Olver I, Van Cutsem E, Svensson C, Seitz JF, Harper P, Kerr D, Perez-Manga G, Azab M, Seymour L, Lowery K and the 'Tomudex' Colorectal Cancer Study Group (1995) 'Tomudex' (ZD1694): results of a randomised trial in advanced colorectal cancer demonstrate efficacy and reduced mucositis and leucopenia. *Eur J Cancer* **31**: 1945–1954

Drake JC, Allegra CJ, Moran RG and Johnston PG (1996) Resistance to tomudex (ZD1694): multifactorial in human breast and colon carcinoma cell lines. *Biochem Pharmacol* **51**: 1349–1355

El-Deiry WS, Harper JW, O'Connor PM, Velculescu VE, Canman CE, Jackman J, Pietenpol JA, Burrel M, Hill DE, Wang Y, Wiman KG, Mercer WE, Kastan MB, Kohn KW, Elledge SJ, Knzler KW and Vogelstein B (1994) WAFl/CIP1 is induced in p53-mediated G1 arrest and apoptosis. *Cancer Res* **54**: 1169–1174

Fisher TC, Milner AE, Gregory CD, Jackman AL, Aherne GW, Hartley JA, Dive C and Hickman JA (1993) bcl-2 modulation of apoptosis induced by anticancer drugs: resistance to thymidylate stress is independent of classical resistance pathways. *Cancer Res* **53**: 3321–3326

Freemantle SJ, Jackman AL, Kelland LR, Calvert AH and Lunec J (1995) Molecular characterisation of two cell lines selected for resistance to the folate-based thymidylate synthase inhibitor, ZD1694. *Br J Cancer* **71**: 925–930

Gartenhaus RB, Wang P and Hoffmann P (1996) Induction of the WAFl/CIP1 protein and apoptosis in human T-cell leukemia virus type I-transformed lymphocytes after treatment with adriamycin by using a p53-independent pathway. *Proc Natl Acad Sci USA* **93**: 265–268

Ikeda MA, Jakoi L and Nevins JR (1996) A unique role for the Rb protein in controlling E2F accumulation during cell growth and differentiation. *Proc Natl Acad Sci USA* **93**: 3215–3220

Jackman AL and Calvert AH (1995) Folate-based thymidylate synthase inhibitors as anticancer drugs. *Ann Oncol* **6**: 871–881

Jackman AL, Marshaman PR, Moran RG, Kimbell R, O'Connor BM, Hughes LR and Calvert AH (1991*a*) Thymidilate synthase inhibitors: the in vitro activity of a series of heterocyclic benzoyl ring modified 2-desamino-2-methyl-$N^{10}$-substituted-5,8-dideazafolates. *Adv Enzyme Regulation* **31**: 13–27

Jackman AL, Taylor GA, Gibson W, Kimbell R, Brown M, Calvert AH, Judson IR and Hughes LR (1991*b*) ICI1694, a quinazoline antifolate thymidylate synthase inhibitor that is a potent inhibitor of L1210 tumor cell growth in vitro and in vivo: a new agent for clinical study. *Cancer Res* **51**: 5579–5586

Jackman AL, Gibson W, Brown M, Kimbell R and Boyle FT (1993) The role of the reduced-folate carrier and metabolism to intracellular polyglutamates for the activity of ICI1694. In: *Proceedings of the International Symposium on Novel Approaches to Selective Treatments of Human Solid Tumors: Laboratory and*

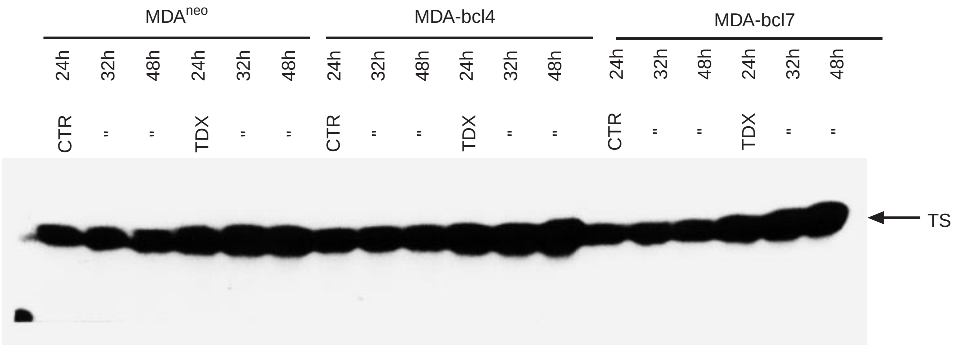

**Figure 8** Effect of TDX on TS expression in MDA$^{neo}$, MDA-bcl4 and MDA-bcl7 cells. Treatments and samples as indicated in Figure 3

© 1999 Cancer Research Campaign

*Clinical Correlation. Advances in Experimental Medicine and Biology*, Rustum YM (ed.) **339**: 265–276. Plenum Press: New York

Jackman AL, Farrugia DC, Gibson W, Kimbell R, Harrap KR, Stephens TC, Azab M and Boyle FT (1995*a*) ZD1694 (tomudex): a new thymidylate synthase inhibitor with activity in colorectal cancer. *Eur J Cancer* **31**: 1277–1282

Jackman AL, Kelland LR, Kimbell R, Brown M, Gibson W, Aherne GW, Hardcastle A and Boyle FT (1995*b*) Mechanisms of acquired resistance to the quinazoline thymidylate synthase inhibitor ZD1694 (tomudex) in one mouse and three human cell lines. *Br J Cancer* **71**: 914–924

Judson IR (1997) ‘Tomudex’ (raltitrexed) development: preclinical, phase I and II studies. *Anti-Cancer Drugs* **8**: S5–S9

Kelland JR, Kimbell R, Hardcastle A, Aherne GW and Jackman AL (1995) Relationships between resistance to cisplatin and antifolates in sensitive and resistant tumour cell lines. *Eur J Cancer* **31**: 981–986

Kinsella AR, Smith D and Pickard M (1997) Resistance to chemotherapeutic antimetabolites: a function of salvage pathway involvement and cellular response to DNA damage. *Br J Cancer* **75**: 935–945

Kroemer G (1997) The proto-oncogene bcl-2 and its role in regulating apoptosis. *Nature Med* **3**: 614–620

Li WW, Fan J, Hochhauser D and Bertino JR (1997) Overexpression of $p21^{wafl}$ leads to increased inhibition of E2F-1 phosphorylation and sensitivity to anticancer drugs in retinoblastoma-negative human sarcoma cells. *Cancer Res* **57**: 2193–2199

Lu K, Yin M-B, McGuire JJ, Bonmassar E and Rustum YM (1995) Mechanisms of resistance to *N*-[5-[*N*-(3,4-dihydro-2-methyl-4-oxoquinazolin-6-ylmethyl)-*N*-methylamino]-2-thenoyl]-L-glutamic acid (ZD1694), a folate-based thymidylate synthase inhibitor, in the HCT-8 human ileocecal adenocarcinoma cell line. *Biochem Pharmacol* **50**: 391–398

O’Connor PM, Jackman J, Bae I, Myers TG, Fan S, Mutoh M, Scudiero DA, Monks A, Sausville EA, Weinstein JN, Friend S, Fornace AJ and Kohn KW (1997) Characterization of the p53 tumor suppressor pathway in cell lines of the National Cancer Institute Anticancer Drug Screen and correlation with the growth-inhibitory potency of 123 anticancer agents. *Cancer Res* **57**: 4285–4300

Panadero A, Yin M-B, Voigt W and Rustum YM (1995) Contrasting patterns of DNA fragmentation induced by thymidylate synthase inhibitors, ZD1694 and AG-331. *Oncol Res* **7**: 73–81

Pinard M-F, Jolivet J, Ratnam M, Kathmann I, Molthoff C, Westerhof R, Schornagel JH and Jansen G (1996) Functional aspects of membrane folate receptors in human breast cancer cells with transport-related resistance to methotrexate. *Cancer Chemother Pharmacol* **38**: 281–288

Prabhu NS, Blagosklonny MV, Zeng YX, Hu GS, Waldman T and El-Deiry WS (1996) Suppression of cancer cell growth by adenovirus expressing p21WAFl/CIP1 deficient in PCNA interaction. *Clin Cancer Res* **2**: 1221–1229

Russo T, Zambrano N, Esposito F, Ammendola R, Cimino F, Fiscella M, Jackman J, O’Connor PM, Anderson CW and Appella E (1995) A p53-independent pathway for activation of WAFl/CIP1 expression following oxidative stress. *J Biol Chem* **270**: 29386–29391

Rustum YM, Harstrick A, Cao S, Vanhoefer U, Yin M-B, Wilke H and Seeber S (1997) Thymidylate synthase inhibitors in cancer therapy: direct and indirect inhibitors. *J Clin Oncol* **15**: 389–400

Sheikh MS, Li X-S, Chen J-C, Shao Z-M, Ordonez JV and Fontana JA (1994) Mechanisms of regulation of WAFl/CIP1 gene expression in human breast carcinoma: role of p53-dependent and independent signal transduction pathways. *Oncogene* **9**: 3407–3415

Sheikh MS, Rocheort H and Farcia M (1995) Overexpression of p21 WAFl/CIP1 induces growth arrest, giant cell formation and apoptosis in human breast carcinoma cell lines. *Oncogene* **11**: 1899–1905

Sherr CJ (1996) Cancer cell cycles. *Science* **274**: 1672–1677

Silvestrini R, Zaffaroni N, Costa A, Orlandi L, Villa R and Hendriks HR (1993) Flunarizine as a modulator of doxorubicin resistance in human colon-adenocarcinoma cells. *Int J Cancer* **55**: 363–639

Smith I, Jones A, Spielmann M, Namer M, Green MD, Bonneterre J, Wander HE, Hatschek T, Wilking N, Zalcberg J, Spiers J and Seymour L (1996) A phase II study in advanced breast cancer: ZD1694 (‘Tomudex’) a novel direct and specific thymidylate synthase inhibitor. *Br J Cancer* **74**: 479–481

Van der Wilt CL, Pinedo HM, Smid K and Peters GJ (1992) Elevation of thymidylate synthase following 5-fluorouracil treatment is prevented by the addition of leucovorin in murine colon tumors. *Cancer Res* **52**: 4922–4928

Van Cutsem E (1997) Future developments with ‘tomudex’ (raltitrexed). *Anti-Cancer Drug* **8**: S33–S38

Westerhof GR, Rijnboutt S, Schornagel JH, Pinedo HM, Peters GJ and Jansen G (1995) Functional activity of the reduced folate carrier in KB, MA104, and IGROV-I cells expressing folate-binding protein. *Cancer Res* **55**: 3795–3802

Xiong Y, Hannon GJ, Zhang H, Casso D, Kobayashi R and Beach D (1993) p21 is a universal inhibitor of cyclin kinases. *Nature* **366**: 701–704

 © 1999 Cancer Research Campaign
